# Influence of BAK-Preserved Prostaglandin Analog Treatment on the Ocular Surface Health in Patients with Newly Diagnosed Primary Open-Angle Glaucoma

**DOI:** 10.1155/2013/603782

**Published:** 2013-07-18

**Authors:** Martina Tomić, Snježana Kaštelan, Kata Metež Soldo, Jasminka Salopek-Rabatić

**Affiliations:** ^1^Department of Ophthalmology, University Clinic Vuk Vrhovac, Clinical Hospital Merkur, Zajčeva 19, 10000 Zagreb, Croatia; ^2^Department of Ophthalmology, Clinical Hospital Dubrava, Avenija Gojka Šuška 6, 10000 Zagreb, Croatia; ^3^Department of Ophthalmology, Clinical Hospital Sveti Duh, Sveti Duh 64, 10000 Zagreb, Croatia

## Abstract

*Purpose*. Primary open-angle glaucoma (POAG), a chronic, degenerative optic neuropathy, requires persistent decrease of intraocular pressure so as to prevent visual impairment and blindness. However, long-term use of topical ocular medications may affect ocular surface health. Purpose of this study was to evaluate the influence of BAK-preserved prostaglandin analog treatment on the ocular surface health in patients with newly diagnosed POAG. *Methods*. 40 newly diagnosed POAG patients were included in this prospective study. Intraocular pressure (IOP), tear break-up time (TBUT), and ocular surface disease index (OSDI) were assessed at baseline and 3-month after starting treatment with BAK-preserved travoprost 0.004%. *Results*. IOP decreased in all patients from baseline to 3-month final visit (23.80 ± 1.73 mmHg versus 16.78 ± 1.27 mmHg; *P* < 0.001). Mean TBUT decreased from 11.70 ± 1.86 seconds at baseline to 8.30 ± 1.29 seconds at 3-month final visit (<0.001). Mean OSDI score increased from 31.63 ± 18.48 to 44.41 ± 16.48 (*P* < 0.001). *Conclusions*. This study showed that BAK-preserved travoprost 0.004% is an effective medication in newly diagnosed POAG patients, but its long-term use may negatively influence ocular surface health by disrupting the tear film stability. Further studies are needed to better understand the clinical effects of different preservative types and concentrations on the ocular surface.

## 1. Introduction

Glaucoma is a group of chronic degenerative diseases of optic nerve head, second leading cause of global blindness, and the leading cause of irreversible visual loss in the adult population worldwide, estimated to have affected 60.5 million people in 2010 and projected to affect 79.6 million by 2020 [1, 2]. It is characterized by the loss of retinal nerve fiber tissues, recognized clinically as visual field defect and loss of the neuroretinal rim of the optic nerve head, termed glaucomatous optic neuropathy (GON). The most prevalent glaucoma form is open-angle glaucoma (OAG), with almost 45 million sufferers worldwide. This number is expected to increase to 58.5 million by 2020 [2, 3]. 

Many clinical researches have shown that damage to the optic nerve in glaucoma is associated with elevated intraocular pressure (IOP) [4, 5]. Decreasing IOP reduces both the incidence of glaucoma in individuals without optic nerve damage and the rate of new damage in individuals with glaucoma. Medical and surgical treatments that decrease IOP thus may prevent or slow the progression of visual impairment and blindness [6]. 

Medical treatment that decreases aqueous humor production or increases outflow is most often the initial treatment for open-angle glaucoma patients [7]. These patients require lifelong treatment and follow-up care to preserve vision, so long-term patient compliance and medication persistence are essential; otherwise they risk developing elevated IOP levels and additional progressing visual impairment to blindness. Compliance and persistence depend on many factors, including patient understanding of the importance of taking their medication over the long term, patient satisfaction with medication, ease of medication administration, and medication costs, although the most important factors are local and systemic side effects.

 Among topical ophthalmic medications, prostaglandin analogs (PGA) show several advantages over other medical treatments and are nowadays the initial medications of choice [8]. However, some prostaglandin-treated patients experience conjunctival hyperaemia or ocular discomfort including burning, stinging, pain, dry eye, or foreign body sensation and these conditions are of concern because these side effects may have a negative effect on whether the patient takes the medication as directed (compliance) and/or continues to use the medication over time (persistence) [9]. Many prostaglandin analogs contain preservatives which have been associated with an increase in the prevalence of ocular signs and symptoms [10]. The most commonly used preservative is benzalkonium chloride (BAK) [11]. 

BAK is a quaternary ammonium compound whose antimicrobial activity arises from its ability to disrupt cell membranes and potentiate cell death. Along with its antimicrobial activity, BAK has a high affinity for membrane proteins and may accumulate in ocular tissues, inducing cell toxicity and/or cell death in a dose- and time-dependent manner. BAK-induced changes in corneal and conjunctival cell membranes may manifest as symptomatic ocular surface disease (OSD) in medically treated glaucoma patients. 

OSD presents a group of disorders that affect various components of the ocular surface. The common consequence of ocular surface disease is dysfunction of the ocular tear film and/or the integrity of the ocular surface. These changes may result in a wide range of ophthalmic symptoms and signs including discomfort, burning, fatigue, fluctuating visual acuity, ulceration, and scarring of the ocular surface. Although OSD is seen in nearly 15% of general population [12, 13], it has been reported to occur in 48% to 59% of patients with medically treated glaucoma [14]. A higher incidence and severity of OSD has been reported in patients who received multiple BAK-preserved medications concomitantly than in patients who were treated with only one BAK-preserved medication.

The purpose of this study was to evaluate the influence of BAK-preserved prostaglandin analog treatment on the ocular surface health in patients with newly diagnosed POAG. We aimed to quantify the changes in the IOP level, tear break-up time (TBUT), and ocular surface disease index (OSDI) after starting treatment with BAK-preserved travoprost 0.004%.

## 2. Patients and Methods

This was a prospective study performed in collaboration of the ophthalmology departments of three Croatian hospitals in accordance with the Declaration of Helsinki and approved by the ethics committee of each Hospital. The patients included in the study received both written and oral information about the study and signed a written informed consent. 

### 2.1. Patients

40 newly diagnosed primary open-angle glaucoma (POAG) patients were included in the study. POAG was defined as characteristic GON and visual field loss, with IOP > 21 mmHg on two separate occasions and a widely openangle. Typical GON was defined as a vertical cup-to-disc ratio (C/D) greater than 0.5, asymmetry of the C/D > 0.2 between eyes, presence of localized RNFL defects, optic disc haemorrhages, and/or neuroretinal rim defects in the absence of any other abnormalities that could explain such findings. Assessment of GON was based on stereoscopic indirect slit lamp fundus examination using noncontact lens (VOLK Super 66) and color fundus photographs (disk field) of both eyes taken with a suitable 45° fundus camera (VISUCAM, Zeiss). A glaucomatous visual field defect in the standard automated perimetry (Octopus 101, G2 program, HAAG-STREIT International) was defined according to the Hodapp classification [15]. Exclusion criteria were previous history of ocular trauma, intraocular surgery, corneal refractive surgery, wearing contact lenses, or having clinically significant ocular surface diseases at baseline such as blepharitis or ocular seasonal allergy, secondary glaucoma, progressive retinal or optic nerve disease, or severe central visual field loss. Patients receiving any ocular medications, oral corticosteroids, or cytostatics, patients with immunologic, infectious inflammatory diseases, and pregnant women were not included in the study.

### 2.2. Methods

Patients who met all inclusion criteria were invited to participate in the study. At the baseline visit, the informed consent form was signed, complete ophthalmic examination including best corrected visual acuity (BCVA), tear break-up time (TBUT), Goldmann applanation tonometry, slit lamp biomicroscopy of the anterior eye segment, binocular indirect slit lamp fundoscopy, and fundus photography after mydriasis with eye drops containing 0.5% tropicamide and 5% phenylephrine was performed, and the ocular surface disease index (OSDI) questionnaire was completed. At the conclusion of the baseline visit, enrolled patients were started glaucoma treatment with BAK-preserved travoprost 0.004% ophthalmic solution (TRAVATAN contains active: travoprost 0.04 mg/mL; preservative: benzalkonium chloride 0.15 mg/mL; inactives: polyoxyl 40 hydrogenated castor oil, tromethamine, boric acid, mannitol, edetate disodium, sodium hydroxide, and/or hydrochloric acid and purified water; S.A. Alcon-Couvreur NV, Puurs, Belgium) once daily in the evening. All patients were required not to use any other topical ophthalmic medications, other than given study medication, for the duration of the study. Patients returned 3-month (90 ± 7 days) after starting glaucoma treatment for the final study visit, which was scheduled at approximately the same time of day as the baseline visit for each patient. At 3 months final visit, an interval medical history was obtained and any side effects were assessed, an ophthalmic examination including slit lamp biomicroscopy of the anterior eye segment, tear break-up time (TBUT), and Goldmann applanation tonometry was performed, and the OSDI questionnaire was completed.

#### 2.2.1. Tear Break-Up Time (TBUT)

This is a method of determining the stability of the tear film and checking for evaporative dry eye. It was obtained by placing 5 *μ*L of 2% preservative free sodium fluorescein (NaFl) to the inferior fornix using a fixed volume micropipette. To carefully mix the NaFl with the tear film, the patients were instructed to blink three times. The slit lamp was set at a magnification of 16× using cobalt blue illumination and a stopwatch was used to time the occurrence of the first break in the fluorescein-stained tear film. The timer was started immediately after the last blink and stopped at the first break in fluorescein. This was measured three consecutive times and an average of these measurements was used to calculate the final TBUT. Despite the wide variation in TBUT among individual subjects, there is general agreement that a TBUT shorter than 10 seconds reflects tear film instability, whereas a TBUT shorter than 5 seconds is a marker of definite dry eye [16]. 

#### 2.2.2. Ocular Surface Disease Index (OSDI) Questionnaire

This is a confirmed, self-administered instrument for assessing the presence and severity of OSD symptoms [17]. The OSDI questionnaire includes 12 questions about the patient's past-week experience with ocular symptoms, vision-related functioning, and environmental triggers [17, 18]. Questions assessed whether patients had eyes that felt gritty, painful, sore, or sensitive to light; whether they had blurred or poor vision; whether they experienced limitations with reading, driving at night, watching television, or working with a computer or bank machine; and whether their eyes felt uncomfortable in windy conditions, in areas with low humidity or in air-conditioned places. Answer options for each question were “all of the time” (score = 4), “most of the time” (score = 3), “half of the time” (score = 2), “some of the time” (score = 1), and “none of the time” (score = 0) [17]. Questions about vision-related functioning or environmental triggers could also be answered with “not applicable,” in which case that question was not factored into the final score calculation. The total OSDI score was calculated for each patient using the methods described by the OSDI originators [17], as follows:

(1) OSDI  score =(sum  of  scores  for  all  questions  answered)×  25total  number  of  questions  answered.

The final total OSDI score could range from 0 to 100, with the OSDI scores classified as ≤12 = normal, 13–22 = mild OSD, 23–32 = moderate OSD, and ≥33 = severe OSD. 

### 2.3. Statistical Analysis

In each patient the “worse” eye was classified using the baseline IOP and TBUT values and later statistically analyzed. Statistical analysis was performed using Microsoft Excel Program for Windows XP. Results are presented as mean ± standard deviation (SD), numbers and percentages. Differences in distributions of continuous data were determined by Student's *t*-test. Differences in distributions of categorical data were evaluated by chi-square test. *P* value of less than 0.05 was considered statistically significant.

## 3. Results

This study included 40 patients with newly diagnosed primary open-angle glaucoma (15 male, 25 female) with a mean age 53.63 ± 10.37 years (range 37 to 70). All forty patients completed the final evaluation and were included in data analysis. The average best corrected visual acuity (BCVA) of included patients was 0.89 ± 0.15, and the average cup-to-disc ratio (C/D) was 0.52 ± 0.11.  Table 1 presents descriptive statistics of demographic characteristics and basic ophthalmologic parameters of newly diagnosed POAG patients included in the study.

Table 2 presents the effectiveness of BAK-preserved travoprost 0.004% treatment on the intraocular pressure (IOP). The mean IOP decreased from 23.80 ± 1.73 mmHg at baseline to 16.78 ± 1.27 mmHg (29.50%) at 3 months of starting glaucoma treatment (*P* < 0.001). 

All patients indicated they tolerated BAK-preserved travoprost 0.004% quite well. None of them discontinued their topical ophthalmic treatment during the 3-month follow-up period and all responded to their treatment (IOP reduction noted in 100% of patients). Subjective symptoms like ocular discomfort, itching, pain, dry eye, or foreign body sensation occurred at an incidence between 1% and 3%. The most common side effect was hyperemia. Eight patients (20%) complained about mild to moderate degree of hyperemia a week after starting treatment, but only three patients (7.5%) had mild degree of hyperemia at final 3-month visit. Systemic tolerability was also noted and most patients did not report any complications.

**Figure 1:**
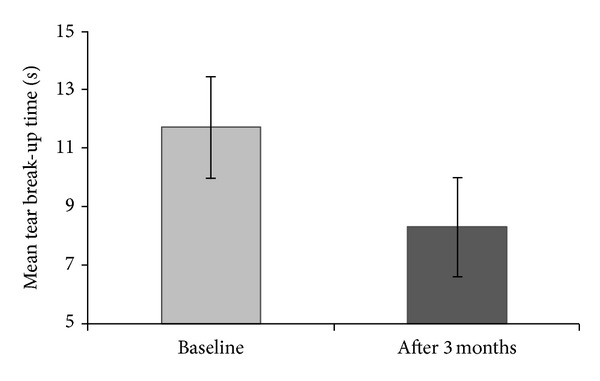
Mean tear break-up time (TBUT) of newly diagnosed POAG patients assessed at baseline and after 3 months.

**Figure 2:**
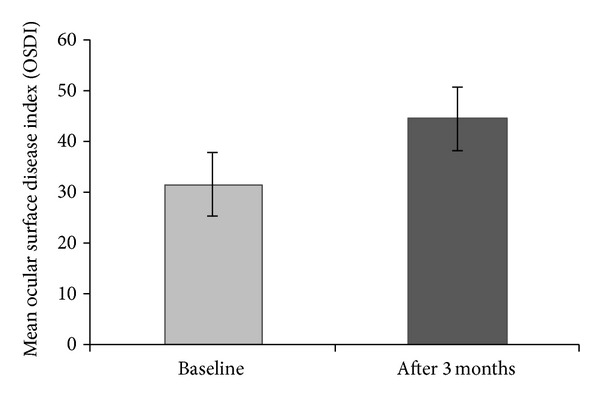
Mean ocular surface disease index (OSDI) of newly diagnosed POAG patients assessed at baseline and after 3 months.

The mean TBUT in enrolled patients at baseline was 11.70 ± 1.86 seconds. At the final visit, 3 months after starting treatment with BAK-preserved travoprost 0.004%, the mean TBUT decreased to 8.30 ± 1.29 seconds (< 0.001), as shown in Table 3 and Figure 1. The mean OSDI increased from moderate category at baseline to severe category at 3 months after initialing glaucoma treatment with BAK-preserved travoprost 0.004% ophthalmic solution (31.63 ± 18.48 versus 44.41 ± 16.48; *P* < 0.001) (Table 3, Figure 2). 

**Figure 3:**
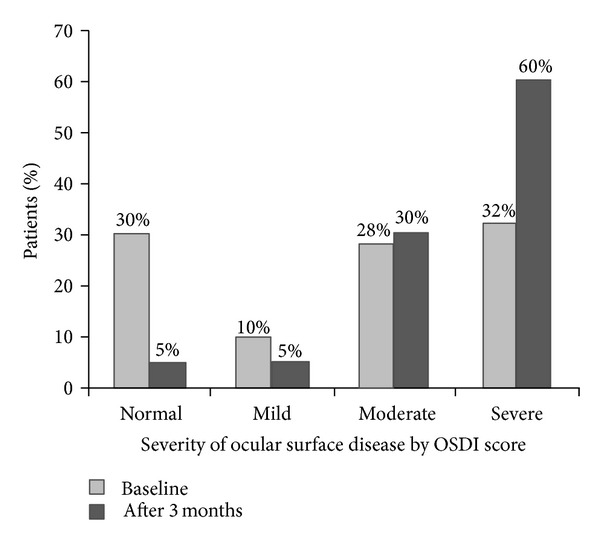
Percentage of newly diagnosed POAG patients with ocular surface disease index (OSDI) scores indicating normal ocular surface or the presence of mild, moderate, or severe ocular surface disease assessed at baseline and after 3 months.

The percentage of patients within each OSDI category (normal ocular surface, mild, moderate, and severe ocular surface disease) were changed from baseline to final visit at 3 months after starting glaucoma treatment, as shown in Figure 3. 

Statistically significant changes were observed in the normal OSDI category (*P* < 0.001) and in the severe OSDI category (*P *= 0.025), as shown in Table 4. 

## 4. Discussion 

The results of this prospective study demonstrate that travoprost 0.004% ophthalmic solution dosed once daily in the evening provides a good intraocular pressure control. It decreased IOP in our patients by 29.50%, from 23.80 ± 1.73 mmHg at baseline to 16.78 ± 1.27 mmHg at 3 months after starting treatment (*P* < 0.001). Our results are in accordance with the results of previous studies. Cantor et al. reported IOP reduction by bimatoprost 0.03% and travoprost 0.004% in patients with open-angle glaucoma or ocular hypertension in the range of 34% to 36% and 19% to 29%, respectively [19]. Meta-analysis of randomized clinical trials reported reduction of IOP in the range of 28% to 31% by latanoprost 0.005%, 29% to 31% by travoprost 0.004% and 28% to 33% by bimatoprost 0.03% [8]. Major clinical glaucoma trials observed the clinical significance of a 30% IOP reduction [20–22]. The sustained 30% IOP reduction from baseline that can be achieved with PGA monotherapy is a reasonable therapeutic target for most patients with open-angle glaucoma or ocular hypertension, while patients with higher IOP, more advanced disease, or high-risk factors such as pseudoexfoliation require greater IOP reductions to achieve disease stability.

Among the 40 patients included in our study, BAK-preserved travoprost 0.004% ophthalmic solution was generally well tolerated, and most side effects were mild to moderate in severity and required no intervention. Ocular hyperemia was the most common side effect. Eight patients (20%) complained about mild to moderate degree of hyperemia a week after starting treatment, but only three patients (7.5%) had mild degree of hyperemia at final 3-month visit. Other common side effects including ocular discomfort, itching, pain, dry eye, or foreign body sensation were rare and occurred at an incidence of 1% to 3%, respectively. None of systemic side effects was reported among patients in our study. Similar results were found in other previous studies [23, 24]. Dubiner and Noecker in their integrated analysis of comprised data from seven peer-reviewed, published, prospective randomized trials reported the rate of physician-reported ocular hyperemia of 38.8% (232/598 patients) and the rate of patient-reported ocular hyperemia of 8.5% (91/1071 patients). The incidence of ocular itching, discomfort, pain, dry eye, and foreign body sensation reported in their analysis was between 1% and 5% [23]. 

Many experimental and clinical studies have shown that the long-term use of topical ophthalmic medication, especially those containing benzalkonium chloride (BAK) as a preservative, may induce changes of the ocular surface, tear film instability, epithelial apoptosis, conjunctival inflammation, fibroblast proliferation, corneal microvilli loss, and goblet cell loss in the conjunctival epithelium [25–27]. Changes of the tear film stability and increase in inflammation caused by BAK often result in a new developing of dry eye, or in a worsening of symptoms in patients with preexisting dry eye what could negatively influence the long-term use of topical ophthalmic medication and consequently the success of glaucoma treatment [28, 29]. Additionally, these ocular surface changes caused by BAK may decrease the success rate of glaucoma surgery [30]. 

As assumed, we found a significantly lower mean TBUT in our patients 3 months after starting treatment with BAK-preserved travoprost 0.004% ophthalmic solution (11.70 ± 1.86 seconds versus 8.30 ± 1.29 seconds; <0.001). According to general guidelines for TBUT [16], the category of the tear film stability in our patients decreased from normal at baseline to tear film instability at 3 months after starting glaucoma treatment. We observed also a significant increase of the mean OSDI score, from moderate category at baseline to severe category at 3 months after initialing glaucoma treatment with BAK-preserved travoprost 0.004% ophthalmic solution (31.63 ± 18.48 versus 44.41 ± 16.48; *P* < 0.001). The percentage of patients within each OSDI category (normal ocular surface, mild, moderate, and severe ocular surface disease) were changed from baseline to final visit at 3 months after starting treatment. Significant changes were observed in the normal OSDI category (*P* < 0.001) and in the severe OSDI category (*P* = 0.025). 

Some previous studies have found similar results [31, 32]. Crichton et al. reported tear film instability in patients with open-angle glaucoma and ocular hypertension after 12-week treatment with different preservative prostaglandin analogs, but with no differences in TBUT between the medication groups (TBUT in seconds: bimatoprost with 0.02% BAK 9.7 ± 5.7; latanoprost with 0.02% BAK 9.3 ± 4.0; *P* = 0.379) [31]. Horsley and Kahook observed the mean TBUT of 2.02 ± 0.71 seconds and the mean OSDI of 26.31 ± 8.25 in open-angle glaucoma patients treated with BAK 0.02%-preserved latanoprost [32]. Ammar et al. found that BAK has significant in-vitro cytotoxicity to cultured ocular epithelial cells. This toxicity of the prostaglandin analogs latanoprost, tafluprost and travoprost preserved with BAK was similar to the toxicity observed in their respective BAK concentrations [33]. Broadway et al. assessed that a significant in vitro cytotoxicity of topical antiglaucoma medications is a result of an increase in inflammation, as presented by significant decrease in goblet cells, increase in pale cells, macrophages, and lymphocytes within the epithelium, and increase in fibroblasts, macrophages, mast cells, and lymphocytes in the substantia propria [29]. Contradictory to previous findings, Schwartz et al. reported the results of the retrospective analysis of three large prescription databases suggesting that open-angle glaucoma and ocular hypotensive patients newly treated with BAK-preserved latanoprost were not significantly likely to develop dry eye, ocular infection, or ocular surface disease as evidenced by additional coding for these disorders during the first year of treatment [34]. They emphasized the importance of adding preservatives to ophthalmic preparations that are instilled multiple times in order to control microbial growth and to prevent the consequences associated with the use of contaminated solutions. Instillation of contaminated eye preparations is a significant risk factor for serious infections including infectious keratitis [35]. 

## 5. Conclusion

BAK-preserved travoprost 0.004% ophthalmic solution dosed once daily in the evening in patients with newly diagnosed primary open-angle glaucoma is effective providing a good intraocular pressure control. It is well tolerated, associated with few side effects which are mild in severity and require neither intervention nor disruption of treatment. Among side effects the most common is ocular hyperaemia. However, its long-term use may negatively influence ocular surface health in patients presented by decreasing in the tear break-up time and increasing in the ocular surface disease index score. 

Therefore, while choosing the medication for glaucoma or ocular hypertension, both the efficacy and the tolerability of medication should be considered, especially in patients who already have ocular surface disease symptoms and clinical signs or who are at high risk of developing them due to use of BAK-preserved medication.

As glaucoma is the leading cause of irreversible blindness worldwide with prevalence being expected to increase in the upcoming years and ocular surface disease being often seen in elderly population and in patients with medically treated glaucoma, further investigations are needed for testing and improving alternative preservative systems in glaucoma medications aimed at preventing the progression of pathologic process and maximizing the safety profiles as a means of ensuring patients compliance and persistence.

## Figures and Tables

**Table 1:** Demographic characteristics and basic ophthalmologic parameters of newly diagnosed POAG patients (*n* = 40) included in the study.

Parameter	New diagnosed POAG patients (*n* = 40)
Age (years)*	53.63 ± 10.37
Sex (m/f)**	15 (37.5%)/25 (62.5%)
BCVA (decimal)*	0.89 ± 0.15
C/D (decimal)*	0.52 ± 0.11

*Mean ± SD; **n (percentage).

POAG: primary open-angle glaucoma; BCVA: best corrected visual acuity; C/D: cup-to-disc ratio.

**Table 2:** Intraocular pressure at baseline and 3 months after starting treatment with BAK-preserved travoprost 0.004% in newly diagnosed POAG patients (*n* = 40) included in the study.

Parameter	Baseline	After 3 months	*P*
IOP (mmHg)*	23.80 ± 1.73	16.78 ± 1.27	<0.001

*Mean ± SD.

POAG: primary open-angle glaucoma; IOP: intraocular pressure.

**Table 3:** TBUT and OSDI at baseline and 3 months after starting treatment with BAK-preserved travoprost 0.004% in newly diagnosed POAG patients (*n* = 40) included in the study.

Parameter	Baseline	After 3 months	*P*
TBUT (sec)*	11.70 ± 1.86	8.30 ± 1.29	<0.001
OSDI*	31.63 ± 18.48	44.41 ± 16.48	<0.001

*Mean ± SD.

IOP: intraocular pressure; TBUT: tear break-up time; OSDI: ocular surface disease index.

**Table 4:** OSDI category at baseline and 3 months after starting treatment with BAK-preserved travoprost 0.004% in newly diagnosed POAG patients (*n* = 40) included in the study.

OSDI category	Baseline	After 3 months	*P*
Normal*	12 (30%)	2 (5%)	<0.001
Mild*	4 (10%)	2 (5%)	0.157
Moderate*	11 (28%)	12 (30%)	0.773
Severe*	13 (32%)	24 (60%)	0.025

*n (percentage).

POAG: primary open-angle glaucoma; OSDI: ocular surface disease index.
